# The Peanut (*Arachis hypogaea* L.) Gene *AhLPAT2* Increases the Lipid Content of Transgenic Arabidopsis Seeds

**DOI:** 10.1371/journal.pone.0136170

**Published:** 2015-08-24

**Authors:** Silong Chen, Yong Lei, Xian Xu, Jiaquan Huang, Huifang Jiang, Jin Wang, Zengshu Cheng, Jianan Zhang, Yahui Song, Boshou Liao, Yurong Li

**Affiliations:** 1 Hebei Provincial Laboratory of Crop Genetics and Breeding, Cereal and Oil Crop Institute, HebeiAcademy of Agricultural and Forestry Science, Shijiazhuang, China; 2 Key Laboratory of Biology and the Genetic Improvement of Oil Crops, Ministry of Agriculture, Oil Crops Research Institute of the ChineseAcademy of Agricultural Sciences, Wuhan, China; University Paris South, FRANCE

## Abstract

Lysophosphatidic acid acyltransferase (LPAT), which converts lysophosphatidic acid (LPA) to phosphatidic acid (PA), catalyzes the addition of fatty acyl moieties to the *sn*-2 position of the LPA glycerol backbone in triacylglycerol (TAG) biosynthesis. We recently reported the cloning and temporal-spatial expression of a peanut (*Arachis hypogaea*) *AhLPAT2*gene, showing that an increase in *AhLPAT2* transcript levels was closely correlated with an increase in seed oil levels. However, the function of the enzyme encoded by the *AhLPAT2* gene remains unclear. Here, we report that *AhLPAT2* transcript levels were consistently higher in the seeds of a high-oil cultivar than in those of a low-oil cultivar across different seed developmental stages. Seed-specific overexpression of *AhLPAT2* in Arabidopsis results in a higher percentage of oil in the seeds and greater-than-average seed weight in the transgenic plants compared with the wild-type plants, leading to a significant increase in total oil yield per plant. The total fatty acid (FA) content and the proportion of unsaturated FAs also increased. In the developing siliques of *AhLPAT2*-overexpressing plants, the expression levels of genes encoding crucial enzymes involved in *de novo* FA synthesis, acetyl-CoA subunit (*AtBCCP2*) and acyl carrier protein 1 (*AtACP1*) were elevated. *AhLPAT2* overexpression also promoted the expression of several key genes related to TAG assembly, sucrose metabolism, and glycolysis. These results demonstrate that the expression of *AhLPAT2* plays an important role in glycerolipid production in peanuts.

## Introduction

Peanuts are one of the largest and most important edible oil-producing crops in the world, surpassed only by soybean and rapeseed in planting area during the last five years[1]; they are used to produce a large percentage of the vegetable oil of many countries. Peanut oil is of high nutritional value; it has high concentrations of unsaturated C18 fatty acids (FAs, approximately 80%) [2], much of which (approximately 45%, or 18:1) is oleic acid, a desirable and healthy type of FA that has been implicated in cardiovascular and cerebrovascular health [3]. With rapid increases in its consumption by humans and its industrial use in renewable biomaterial and fuel production, the demand for vegetable oil has increased significantly [4]. Therefore, high oil content is desirable and has been a major goal of breeding and genetic engineering of oil crops including peanut. The oil content in peanut seeds generally ranges from 42% to 52% [5], which is relatively high compared with most other oilseed crops. However, the highest seed oil content in the peanut germplasm is approximately 63% [6]. It is a challenge to substantially increase the oil content of peanut seeds using conventional breeding and genetic engineering.

In oilseed plants, approximately 95% of seed storage oils are composed of triacylglycerols (TAGs), which are primarily synthesized during the seed’s maturation phase. In plants, TAGs play critical roles in diverse physiological processes and in multiple metabolic reactions[7]; in particular, TAGs that are stored in the endosperm support successful seedling establishment after germination [8].Furthermore, TAGs supply approximately 30% to 35%of dietary calories to people in the developed world[9].

In higher plants, TAGs are synthesized from glycerol-3-phosphate (G3P) and FAs. Through many previous studies, the FA and TAG biosynthetic pathways have been well characterized[10].TAG is synthesized *de novo* in several sequential acylation reactions at the *sn*-1-, *sn*-2- and *sn*-3-positions of the G3P backbone with acyl chains from acyl-CoAs through the conventional Kennedy pathway in the endoplasmic reticulum (ER).These reactions are catalyzed by three acyl-CoA-dependent acyltransferases[9].Besides, in some plants, the synthesis of TAG can also be assembled by transacylation of the *sn*-2 FA from phospatidylcholine (PC) onto *sn*-3-position of diacylglycerols(DAG), catalyzed by the phospholipid: diacylglycerol acyltransferase (PDAT) enzyme during seed maturation [11, 12]. Lysophosphatidic acid acyltransferase (LPAT, EC 2.3.1.51) is one of the major TAG synthesis enzymes and catalyzes the second step of TAG formation, controlling the metabolic flow of lysophosphatidic acid (LPA) to phosphatidic acid (PA)in Kennedy pathway[13].

Considerable knowledge has been gained regarding *LPAT* in eukaryotes. Genes that encode LPAT enzymes have been identified in various organisms, such as mammals, yeast and plants[14–20].There are five isolated and annotated *LPAT* genes designated *AtLPAT1–5*in the Arabidopsis genome[21]. They include the plastidial isoenzyme gene (*AtLPAT1*), the ER-located isoenzyme gene (*AtLPAT2*), the male gametophyte isoenzyme gene (*AtLPAT3*), and two less closely related members, *AtLPAT4* and *AtLPAT5*, which encode proteins without *in vitro* detectable LPAT activity. Increasing evidence shows that different *LPAT*s have distinct expression patterns and functions[22]. Three Arabidopsis *LPAT*s (*AtLPAT1*, *AtLPAT2*, and *AtLPAT3*) are essential to normal plant development[21]. Both Arabidopsis *AtLPAT4* and *AtLPAT5* give rise to alternative transcripts and may have higher orders of complexity in PA synthesis[9].It is worth stressing that studies have focused on orthologous *LPAT2*genesfrom various plants, such as *AtLPAT2*[21], rapeseed *LPAT*s[23], *TmLPAT2*[24], and *EpLPAT2*[25], because of their important roles in TAG bioassembly.

Recent results suggest that *LPAT* also plays important roles in lipid production. An expanding line of research shows that the overexpression of *LPAT* in transgenic seeds is associated with increased TAG and total FA content. When the wild-type and mutant forms of the yeast genes *SLC1* (sphingolipid compensation mutant) and *SLC1-1*, which are homologs of ER-localized *LPAT*s in Arabidopsis, were overexpressed in Arabidopsis, rapeseed, and soybean, the TAG content of the seeds increased [26, 27]. Overexpression of rapeseed *LPAT* genes (*BAT1*.*13* and *BAT1*.*15*) with homology to Arabidopsis *AtLPAT2* in Arabidopsis seeds resulted in transgenic seeds with increased mass and TAG content [23]. Collectively, these data suggest that increasing the expression of *LPAT* in seeds may regulate the flow of carbon intermediates into TAG through the Kennedy pathway, resulting in enhanced TAG accumulation [23]. Therefore, *LPAT* could be a potential target gene for increasing seed oil content, serving as a valuable biotechnological tool. Nevertheless, more functional work remains to be done on the *LPAT*s to understand their metabolic roles in TAG accumulation.

In oilseed plants, LPAT activity is a potential bottleneck for the incorporation of acyl-CoA into PA [21, 28]. Previous experimental studies performed on diverse species have revealed that LPAT enzymes have preferences in the utilization of certain FAs as substrates and are commonly considered some of the most stringent acyltransferases regarding acyl-specificity[24]. Biochemical studies on the LPATs from a number of oilseeds suggested that microsomal LPATs have a generalized preference for 16- and 18-carbon monounsaturated FAs over saturated FAs [29, 30].

The specific functions of *LPAT*s in TAG biosynthesis in oil crops vary among different organisms[25, 31]. Despite studies on model organisms that show the potential of *LPAT*s to change FA composition and increase the oil content of seeds, the precise functions of the majority of *LPAT*s remain largely unknown. Therefore, the identification and functional characterization of new *LPAT* genes will provide useful information on the potential roles of *LPAT*s in oil crops such as peanut, potentially contributing to genetic improvement.

In our previous studies, using cDNA library screening, two full-length transcripts encoding the *LPAT*-family genes*AhLPAT2* and *AhLPAT4* were isolated from developing peanut seeds, and their temporal and spatial expression patterns were analyzed [32, 33]. Gene expression analysis during seed development can be used to identify putative candidates involved in the synthesis of seed lipids such as TAG [31, 34–36].Our results demonstrated that*AhLPAT2*is highly similar to typical microsomal LPAT isoforms.*AtLPAT2*was expressed ubiquitously in diverse organs but most highly in the seeds; it has a pattern of expression similar to that of *RcLPAT2*[31], *AtLPAT2*[21], *BAT1*.*13*[23] and *EpLPAT2*[25] but distinct from that of *TmLPAT2*[24]. Moreover, we found that the *AhLPAT2* expression level increases rapidly during the active phase of oil accumulation and then decreases markedly as the seed lipid content plateaus. In addition, *AhLPAT2* transcript abundance positively correlated with seed oil content (p<0.05).Unlike *AhLPAT2*, the *AhLPAT4* gene is highly expressed, but only in the vegetative organs; its transcript level is considerably lower in seeds. The above lines of evidence indicate a correlation between *AhLPAT2* and lipid biosynthesis in peanut seeds. However, this gene’s exact biological function in regulating seed oil accumulation is still unknown. Therefore, further work will be required to investigate the biological function of *AhLPAT2*.

Our objectives in this study were to identify and validate the potential contribution of *AhLPAT2* to lipid accumulation in peanut. We further examined the expression differences of *AhLPAT2* in developing seeds of high-and low-oil peanut cultivars. Then, the subcellular localization of *AhLPAT2* was examined. We used Arabidopsis to survey the molecular function of the *AhLPAT2* gene due to the difficulty of peanut plant transformation. In transgenic Arabidopsis, the effects of overexpression on seed oil content, FA composition and seed yield were analyzed. Our study provides a better understanding of the functional role of *AhLPAT2* in seed oil accumulation. The results demonstrate that seed-specific overexpression of *AhLPAT2* can be employed to increase TAG content and may facilitate improvements in oil production in peanut and other oil crops through genetic manipulation of the *AhLPAT2* gene.

## Materials and Methods

### Plant materials

Seeds of the peanut cultivars Zhonghua12 and Te21 were planted at the Dishang Experimental Station of Food and Oil Crops Research Institute of the Hebei Academy of Agriculture and Forestry Sciences in Shijiazhuang, China(37.56°N, 114.43°E)between May and September.Zhonghua12 is a high-oil cultivar whose seed oil content is approximately 59.4%;Te21 is a low-oil cultivar whose seed oil content is approximately 49.3%[37]. The treatment and collection of developing peanut seeds were performed as described by Yin *et al*.[38], with minor modifications. Seeds at various developmental stages (10, 20, 30, 40, 50, 60, 70 and 80 days after flowering, or DAF) were removed from the immature pods of the two genotypes, frozen in liquid nitrogen, and stored at –80°Cfor RNA isolation and analysis.

*Arabidopsis thaliana* (ecotype Col-0) was used for transformation in this study. The wild-type Col-0 and the transgenic plants were grown under standard conditions as described previously [39]. The Arabidopsis seeds were surface-sterilized in 3% sodium hypochlorite for 20 min and rinsed 10 times in distilled water. The sterilized seeds were then sown on plates containing Murashige & Skoog (MS)germination medium and kept for 3 days at 4°C in the dark to break dormancy. After 1 week, the seedlings were transferred to a pot filled with a mixture of peat/forest soil and vermiculite (1:1, v/v)and kept in the same controlled-environment growth chamber under the following conditions:22°C with a diurnal photoperiod of 16 h light (50μmol/m^2^/s) and 8 h dark per day and 70% relative humidity. For consistency in the reproducibility of the oil content measurements, the transgenic lines were always grown with Col-0 plants in the same chamber at the same time[40].

### The subcellular localization of *AhLPAT2*

The full-length *AhLPAT2*ORF was fused without a stop codon downstream of the constitutive CaMV 35S promoter and upstream of *GFP* (NCBI accession number U87973) in a pEGFP expression vector, creating an AhLPAT2-GFP fusion protein. The coding sequence, including the *Xma*I (5′) and *Bam*HI (3′) restriction sites, was amplified using the primers 5′–TCCCCCGGGATGGGTATTGCAGCTGCTGCT–3′ and 5′–CGCGGATCCGGCTGTTGTTTGACATTCTT–3′. The PCR product and the pEGFP plasmid were cut with *Xma*I and *Bam*HI and ligated together using T4 ligase. The expression of *AhLPAT2* and *GFP* was under control of the CaMV 35S promoter. The sequences of the fusion constructs were verified by sequencing. The recombinant pEGFP AhLPAT2-GFP plasmid and the control pEGFP plasmid were bombarded into 20 to 25 onion epidermal segments using a Helios gene gun (Bio-Rad, Hercules, CA) according to the procedures described in [41, 42].After incubation on MS medium at 25°C for 36 h in the dark, the transformed cells were observed by confocal microscopy (Nikon A1, Japan). DAPI (4′, 6-diamidine-2′-phenylindole dihydrochloride; Roche, Germany) in methanol solution (1 μg/ml) was used to visualize nuclei.

### Construction of an *AhLPAT2* vector for seed-specific expression and plant transformation

The coding region of the *AhLPAT2* gene was amplified using the *AhLPAT2* forward primer 5′–GGCGAGCTCATGGGTATTGCAGCTGCTGCT–3′,which contained a*Sac*I restriction site, and the *AhLPAT2* reverse primer 5′–TCCCCCGGGCTACTGTTGTTTGACATTCTT–3′,which contained an *Xma*I restriction site. The amplified*AhLPAT2* gene with the flanking restriction sites was subcloned into the corresponding sites of the plant transformation vector pBarN in the sense orientation. pBarN was derived from the plant binary vector pCAMBIA-1301(AF234297) [43] by introducing a *Hin*dIII/*Sac*I fragment containing the rapeseed napin promoter [44] and an *Eco*RI/*Hin*dIII fragment containing the NOS terminator [45]. The hygromycin phosphotransferase coding region and its promoter were replaced with a fragment containing the 35S promoter and the*Bar* gene, which confers resistance to glufosinate-ammonium and was used as a selectable marker to screen for transgenic plants. The resulting construct was designated *Napin*:*AhLPAT2*.The construct’s integrity was confirmed by sequencing. The plasmid was transformed into *Agrobacterium tumefaciens* strain GV3101 by electroporation (Gene Pulser II, Bio-Rad, Denmark) and then introduced into wild-type Arabidopsis using the floral dip method [46].

### Selection of transgenic plants

To confirm transgene integration, T0 transgenic seeds were selected on plates containing MS medium supplemented with 100 mg/ml glufosinate. After 7 to 10 d, herbicide-resistant seedlings that had green leaves were identified as T1 transformants, and they were transferred to soil. The presence of the *AhLPAT2* transgene was confirmed by PCR ongenomic DNA isolated from the leaves of the T1 seedlings using the primers NapinHinF (5′–CCCAAGCTTAGCTTTCTTCATCGGTGATTGAT–3′) and AhLPAT2XmaR (5′–TCCCCCGGGCTACTGTTGTTTGACATTCTT–3′). Independent transgenic Arabidopsis lines exhibiting a 3:1 segregation of glufosinate (30μg/ml) resistance in the T2 generation were selected, and homozygous T3 transgenic lines were established. T2 lines exhibiting greateroil deposition and *AhLPAT2* expression than Col-0 were propagated to yield T3 seed lines. The quantitation of *AhLPAT2* expression in transgenic Arabidopsis siliques was performed as described below. Seed oil content and FA analyses were conducted using seeds from the T1, T2 and T3 generations of transgenic Arabidopsis. Additional assays were performed, and data on plant height, the number of siliques per plant, seeds per silique, and silique length were collected from homozygous transgenic lines in the T3 generation.

### Measurement of seed size and 100-seed weight

Arabidopsis seed size was determined as described previously [47]. The seeds from each line were dried in open tubes in a desiccator for 48 h prior to measuring and weighing. Arabidopsis seeds were photographed on white paper using a high-resolution scanner (Canon, Japan). After thresholding and converting the photographs to binary images, seed size was measured using the “Analyze particles” function of ImageJ (National Institutes of Health). The sizes of 40 to 50 seeds per silique per genotype were determined. To determine seed weight, batches of 100 seeds were weighed using a microbalance (Sartorius, Germany).

### Determination of seed oil content and quantitative FA analysis

The oil content ofair-dried peanut seeds at different developmental stages was determined using nuclear magnetic resonance (NMR) analysis as described previously [32]. Seed oil content was expressed as a percentage of dry seed weight. To determine the total oil content and FA composition of the Arabidopsis lines, the mature seeds were measured using gas chromatography (GC)coupled with a flame ionization detector (FID) as described previously[23].Twenty milligrams of Arabidopsis seeds from each line were subjected to direct transmethylation by the addition of 1 ml of 5% (v/v) H_2_SO_4_ in methanol (CH_3_OH)containing 0.2% butylated hydroxytoluene (BHT) in glass tubes with Teflon-lined screw caps. For oil extraction and transmethylation, the samples were heated for 1.5 h to between90°Cand 95°C. FA methyl esters (FAMEs) were extracted with hexane. FAMEs were quantified using GC (Agilent 7890A, CA) on a 0.25 mm × 30 m column with Carbowax (Altech) using helium as a carrier gas and20 ml/min flow, and flame ionization was used for detection. The injector temperature was 250°C, and the detector temperature was 260°C. The injection volume was 1 μl. The oven temperature was maintained at 170°C for 1 min and then ramped to 210°C at 3°C per minute; it was then maintained at this temperature for an additional 8 min. FAMEs were identified by comparing their retention times with those of a standard mixture of FAMEs. One hundred microliters of 2.5 mg/ml heptadecanoic acid (17:0) was used as the internal standard to quantify the individual FAs. Measurements of the total FA content and FA composition are given as micrograms of FA per milligram of dry seeds (μg/mg).

### Seed protein analysis

The concentration of protein was determined using the Bradford method[48]and a Bradford protein assay kit (Tiangen, Beijing) according to the manufacturer’s instructions. Twenty milligrams of seeds were ground in 200 μl of extraction solution buffer. Finally, 75 μl of the supernatant was subjected to ultraviolet spectrophotometer analysis on a SPECORD 205 (Analytik, Jena),and the absorbance values at 595 nm were measured.

### RNA isolation and SYBRGreen-mediated quantitative Real-Time PCR (qRT-PCR)

Total RNA was extracted from developing peanut seeds at the indicated stages using the RNeasy Plant Mini Kit (Qiagen, Hilden, Germany) according to the manufacturer’s instructions. Total RNA was isolated from 100 mg of young siliques from wild-type Col-0 and the transgenic Arabidopsis lines 14 DAF using Trizol Reagent (Invitrogen, San Diego, CA). DNA contamination in the RNA samples was removed with RNase-free DNase.

For qRT-PCR, 1 μg of DNase-treated RNA from each sample was used for reverse transcription. The first-strand cDNA (1 μl) was used as the template for qRT-PCR amplification using SYBR Green Master Mix (TOYOBO, Japan). Triplicate quantitative assays were performed on an iQ 5 Multicolor Real-Time PCR Detection System (Bio-Rad, Hercules, CA) as follows: pre-denaturation at 95°C for 5 min; 40 cycles of 95°C for 15 s, 60°C for 20 s, and 72°C for 20 s; and a final extension at 72°C for 5 min. The gene-specific primers for *AhLPAT2* in the peanut seeds were 5′–GGCTTATTGATTGGTGGGCTGGT–3′ and5′–ACCAACCAATGACCGGCAGAAAC–3′. The gene-specific primers for qRT-PCR on the Arabidopsis samples are listed in S1 Table. The primers were properly optimized, and their efficiency was close to one. *AtActin7* (NM_121018)[49]and *AhUbiquitin*[50] were used as reference genes in Arabidopsis and peanut, respectively, to quantify the relative transcription levels of each target gene. The relative transcript abundance of the target genes in the samples was calculated using the 2^–∆∆CT^ method[51].The final values obtained were a measure of the fold changes in expression for the genes of interest.

### Statistical analysis

All experiments were performed using at least three replicates. Significant differences were determined using paired Student's *t*-tests in the SAS statistical package for Windows v. 9.0 (SAS Institute, NC, USA). Values of p*<*0.05 were considered significant.

## Results

### Identification of candidate gene *AhLPAT2* relevant to lipid accumulation

**Fig 1:**
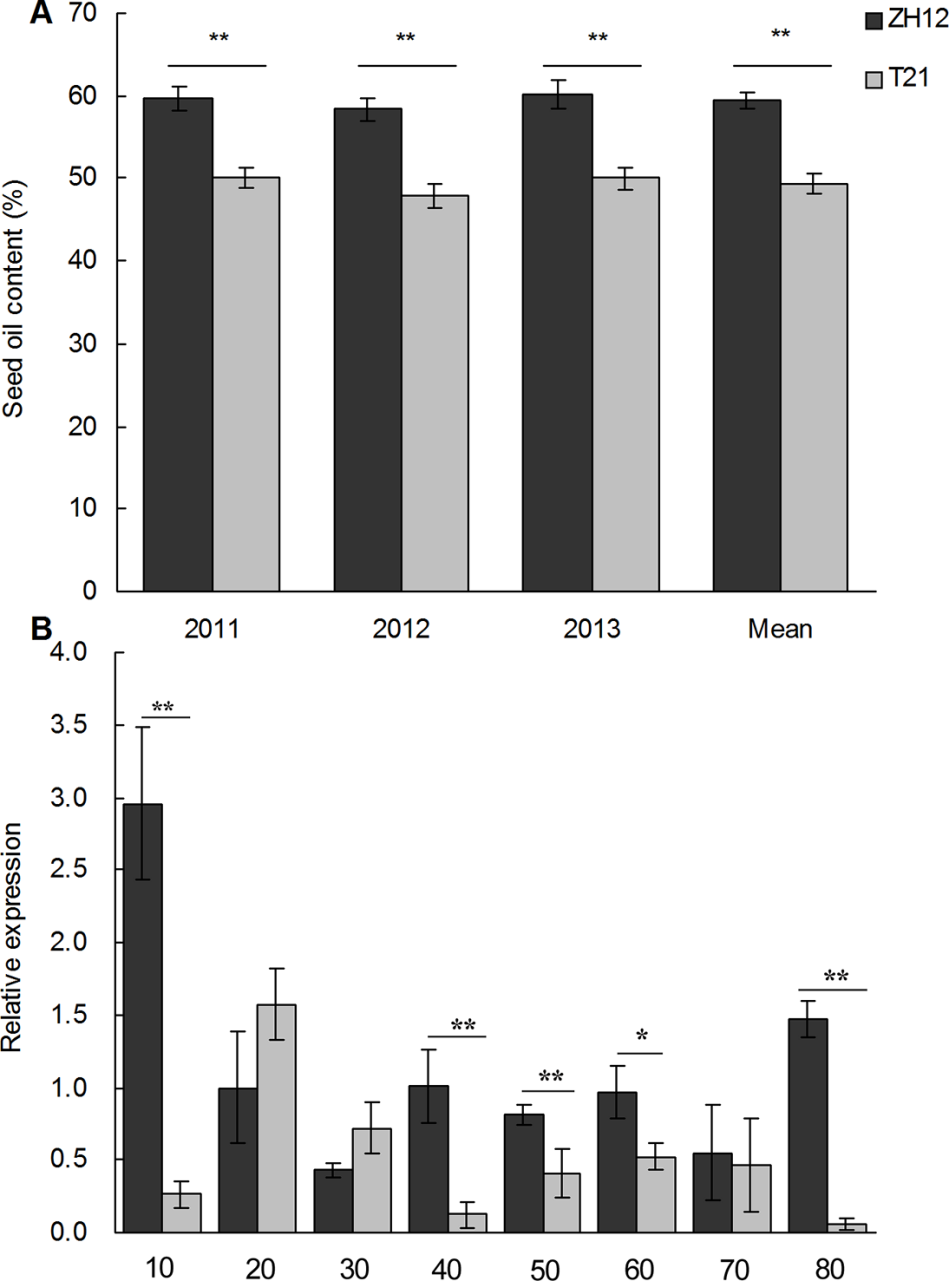
Phenotypic Variation in Seed Oil Content in High-Oil and Low-Oil Peanut Cultivars, Expression Pattern of *AhLPAT2* in Eight Seed Developmental Stages of Them. A. Comparison of seed oil content from high-oil cultivar Zhonghua12 and low-oil Te21. Values are average seed oil content ± SE (*n* = 3). Asterisks indicate a significant difference between the compared pairs at p < 0.01. B. Transcript abundance of *AhLPAT2* in different stages of developing peanut seeds. Total RNA was prepared from developing seeds of Zhonghua12 and Te21 at 10 to 80 DAF. qRT-PCR was performed to compare the overall transcript abundance of *AhLPAT2* in Zhonghua12 and Te21. Gene expression levels are shown relative to the expression of peanut *AhUbiquitin* gene in each sample. Values are means ± SE (*n* = 3).Asterisks indicate significant differences between the wild-type and transgenic lines at p < 0.01 (**) and p < 0.05 (*).

To investigate the expression of genes that possibly participate in oil synthesis in developing peanut seeds, based on experimental results in the field over three years, we selected the high-oil-content peanut cultivar Zhonghua12 and the low-oil-content cultivar Te21. Our results suggest that oil content was stable in both genetic backgrounds over three years. The seed oil content of Zhonghua12 and Te21 differed significantly by more than 10% (p< 0.01) (Fig 1A). Using experimental materials with such large differences in seed oil content, we identified many differentially expressed genes associated with seed oil content in peanut. The *AhLPAT2* gene, isolated from developing peanut seeds by sequencing a full-length-enriched cDNA library and homology-based cloning, was originally identified from gene differential expression analysis using RNAs from different peanut organs and developing seeds at different stages [33].Among the differentially expressed genes chosen and characterized, *AhLPAT2*, a gene encoding a putative LPAT protein, was identified as seed-preferred gene. The expression of *AhLPAT2*had a similar trend to that of oil accumulation in seeds[33].We focused on *AhLPAT2* gene and further dissected its function in lipid accumulation in detail. In the present study, an attempt was made to test and analyze the *AhLPAT2* transcript level at different developmental stages in seeds of Zhonghua12 and Te21.The trend in the *AhLPAT2* expression pattern in developing seeds across the eight stages, from 10 DAF to mature seeds, was similar in the high-oil cultivar Zhonghua12 and the low-oil cultivar Te21. However, the *AhLPAT2* transcript levels were significantly higher in Zhonghua12 seeds than in Te21 seeds at 10, 40, 50, 60, and 80 DAF and especially between 40 and 60 DAF (Fig 1B), when the storage oil accumulated at its maximum rate [38, 52].Taken together with our previous data [33], *AhLPAT2* is associated with seed oil accumulation.

### The subcellular localization of *AhLPAT2*

**Fig 2:**
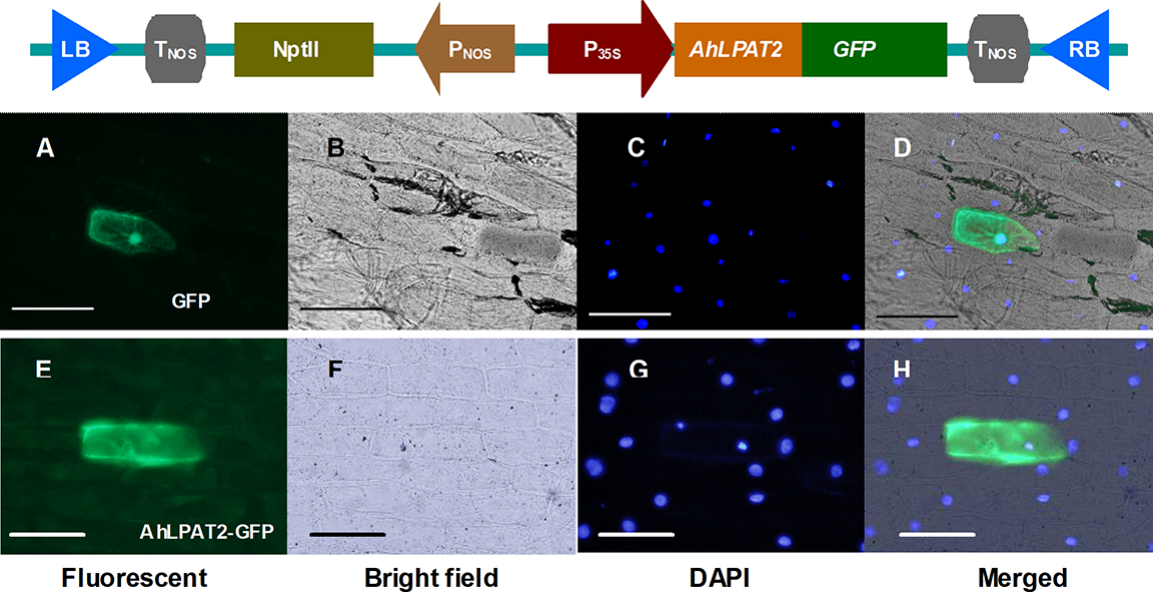
Subcellular Localization of the AhLPAT2–GFP Fusion Protein in Onion Epidermal Cells. The top panels show the expression vector containing *AhLPAT2* and the *GFP* reporter gene. *AhLPAT2* tagged with *GFP* in the C-terminus was inserted into the pEGFP vector between the *Kpn*I and *Xma*I restriction sites. LB, left border; RB, right border; PNOS and TNOS are the promoter and polyadenylation signal of the nopaline synthase gene, respectively; NptII neomycin phosphotransferase II, CaMV 35S promoter. Bottom panels show the expression of *GFP* and *AhLPAT2*-*GFP*. (A) GFP fluorescence of onion epidermal cells expressing *GFP*. (B) Bright field image of (A). (C) Cell nuclei counterstained with DAPI. (D) Merged image of (A), (B) and (C). (E) GFP fluorescence of onion epidermal cells expressing AhLPAT2-GFP. (F) Bright field image of (E). (G) Cell nuclei counterstained with DAPI. (H) Merged image of (E), (F) and (G). Scale bars = 50 μm.

To determine the subcellular localization of *AhLPAT2*, an *AhLPAT2*-*GFP* fusion gene driven by the CaMV 35S promoter was constructed (Fig 2), and *GFP*-tagged *AhLPAT2* was transiently expressed in onion epidermal cells. *GFP* alone generated a strong fluorescent signal that was observed in the cytoplasm and the nucleus (Fig 2A–2D), whereas the signal from *AhLPAT2*-*GFP*was mostly detected in the cytoplasm of transformed cells (Fig 2E–2H). These data indicate that *AhLPAT2*is a cytoplasmic protein.

### The phenotypes of transgenic *AhLPAT2*-overexpressing Arabidopsis plants

**Fig 3:**
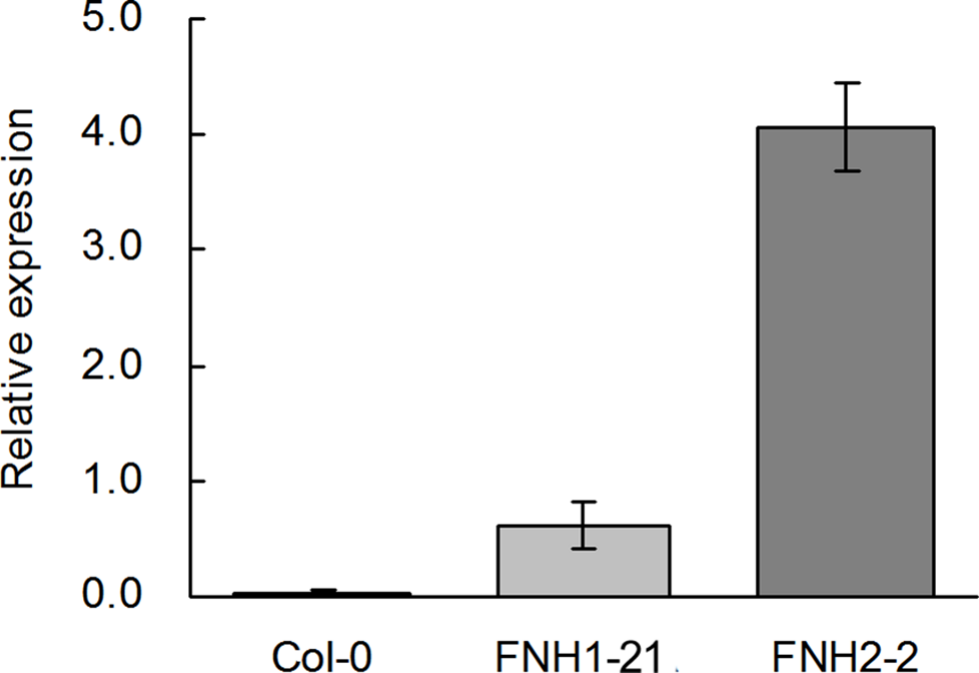
*AhLPAT2* Gene Expression in Young Siliques of the Selected Transgenic Lines Overexpressing *AhLPAT2*. Two transgenic T3 lines, FNH1-21 and FNH2-2, were used. Total RNA was prepared from developing siliques of transgenic lines. Gene expression levels are shown relative to the expression of *AtActin7* in each sample. Values are means ± SE (*n* = 3).

To evaluate the function of*AhLPAT2*, we generated a binary plant transformation vector harboring the *AhLPAT2* coding sequence controlled by the *Napin* promoter and introduced this construct into *Agrobacterium tumefaciens*, which was subsequently used to transform Arabidopsis. The presence of the *AhLAPT2* transgene was confirmed in the T1 seedlings via PCR. Through segregation analysis of the herbicide-resistant transgenic plants, 45 single-copy*AhLPAT2* sense plants were identified. To analyze the effect of the *AhLPAT2* transgene on the lipid phenotypes of the seeds, 11 independent T2 homozygous single-copy *AhLPAT2* plants were randomly selected. After using qRT-PCR with gene-specific primers todetermine the*AhLPAT2* expression levels inthe siliques of the transformed Arabidopsis plants, we selected two independent homozygous lines with relatively high *AhLPAT2*levels (FNH1-21 and FNH2-2)for further analysis. In general, the *AhLPAT2* transcript levels in the developing siliques of line FNH2-2 were higher than in those of line FNH1-21 (Fig 3).

We examined whether *AhLPAT2* overexpression changed any of the plants’ phenotypes. We did not observe any significant difference in plant height or seed development, including mature silique length and seed size, between the transgenic *AhPAT2* lines and wild-type plants(S1 Fig). These results indicated that seed-specific overexpression of*AhLPAT2* does not influence the overall growth and development of Arabidopsis plants.

### Seed weight and yield per transgenic Arabidopsis plant

**Fig 4:**
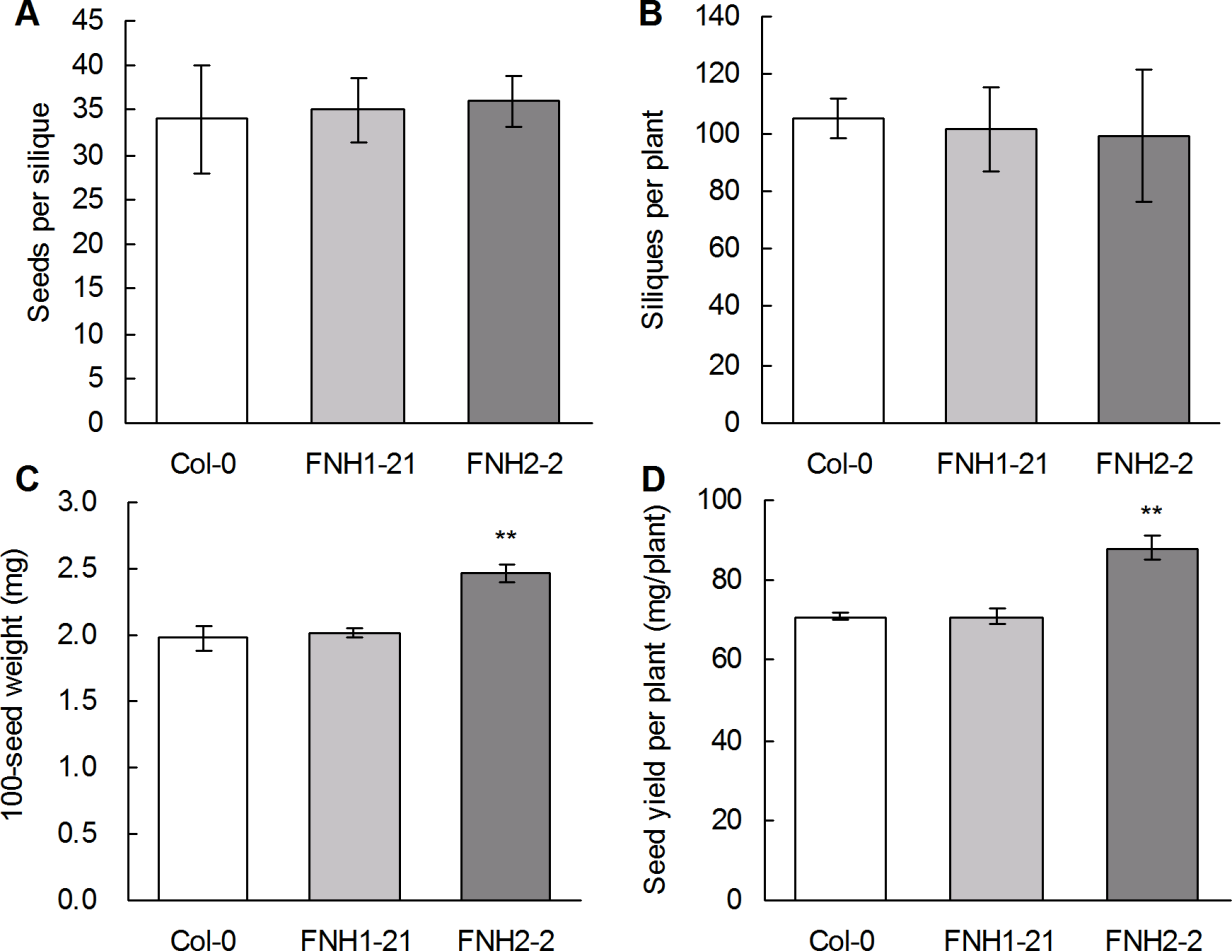
Effect of *AhLPAT2* Overexpression on Seed Storage Reserve Content of Independent Homozygous T3 Lines. A. Seeds per silique. B. Numbers of siliques per plant. C. 100-seed weight. D. Seed yield per plant. Values are means ± SE (*n* = 10). Asterisks indicate significant differences between the wild-type and transgenic lines at p < 0.01 (**).

Because the amount of seed oil produced per plant is dependent on the oil content and the seed yield, and because the seed yield per plant is dependent on seed weight, the number of seeds per silique, and the number of siliques per plant, we examined the effect of *AhLPAT2*overexpression on the seed yield per plant. The number of seeds per silique and the number of siliques per plant were not significantly changed in the two T3 homozygous transgenic lines (Fig 4A and 4B). By contrast, the 100-seed weight was significantly increased in the transgenic FNH2-2 line compared with Col-0 (Fig 4C). As a result, when the increase in the average seed weight of FNH2-2 was taken into account, the average seed yield per plant was 24.4% greater than that of wildtype (Fig 4D). However, the seed weight and yield of FNH1-21 were not severely affected by seed-specific overexpression of *AhLPAT2* than FNH2-2, suggesting that the phenotypes of the transgenic lines derived from different independent transformation event are different.

### The seed oil content of transgenic Arabidopsis

Analysis of 42 transgenic*AhLPAT2* Arabidopsis overexpression plants showed that the seed oil content ranged from 25.5% to 36.0%, with a mean value of 32.2%;this mean is 7.4% higher than that of non-transgenic plants. Thirty-four of the plants had higher seed oil content than the non-transformed seeds (S2A Fig).

We also measured the total lipid content of Col-0 seeds andof seeds from the *AhLPAT2*-overexpressing lines, including the 11 independent T2 lines and the twoT3 homozygous lines. The T2 plants were derived from representative T1 transgenic seeds. The homozygous *Napin*:*AhLPAT2*FNH1 T2 lines exhibited oil content increases ranging from 1.7 to 3.8 percentage points on a mature seed weight basis, representing a net overall increase of 5.5% to 12.2%;the oil content of the FNH2 lines exhibited oil content increases ranging from 1.7 to 6.9 percentage points, representing net overall increases of 5.4% to 22.1% compared with that of the wild-type plants grown in the same growth environment at the same time (S2B Fig).

The average oil contents of the five transgenic FNH1 lines and the six FNH2 lines were 33.7% and 35.5%, respectively, which were significantly different from the average 31.2% oil content of the non-transformed plants (p<0.05).Combining the results from the five FNH1 and six FNH2 lines, the mean seed oil content was 34.6%, which was11.1% greater than the mean of the non-transformed plants (p<0.05; S2B Fig).The homozygous FNH1-21 and FNH2-2 plants were analyzed in the next generation.

**Fig 5:**
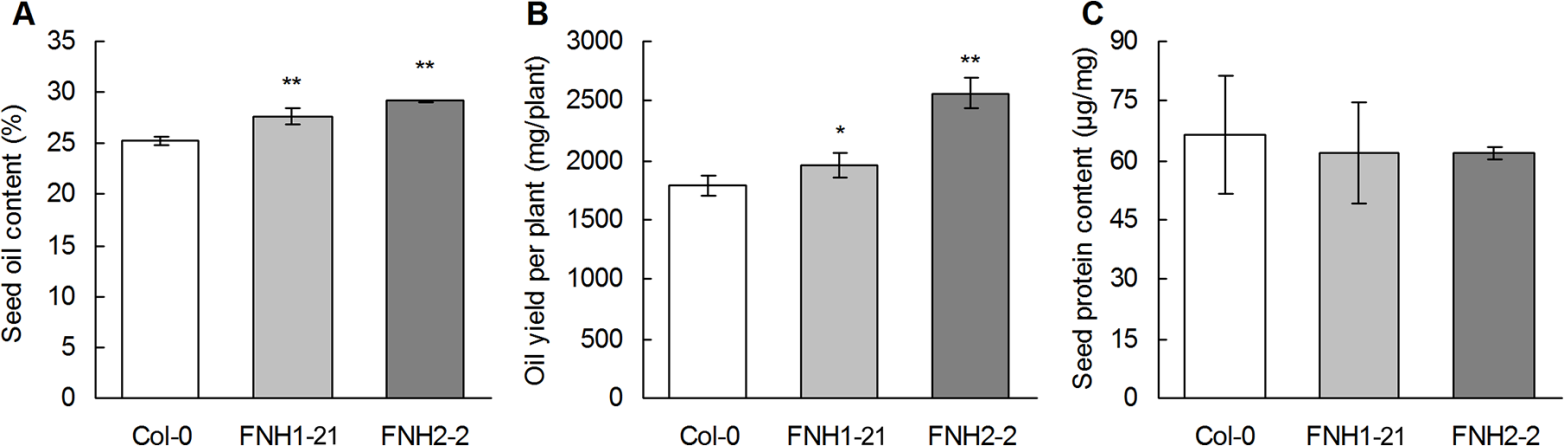
Effect of *AhLPAT2* Overexpression on Seed Oil and Protein Contents of Independent Homozygous T3 Lines. A. Seed oil content as a percentage. B. Oil yield per plant. C. Seed protein content. Values are means ± SE of measurements of individual plants (*n* = 10). Asterisks indicate a significant difference between the wild-type and transgenic lines at p < 0.01 (**)and p < 0.05 (*).

In the T3 homozygous seeds, *AhLPAT2* overexpression caused a significant increase in oil content compared with Col-0 seeds. The oil content in the seeds of the two transgenic Arabidopsis lines (FNH1-21 and FNH2-2)was approximately 28.4%, which was 12.2% higher than that of Col-0 (25.3%; Fig 5A). The T3 homozygous transgenic seeds had an oil content similar to that of the T2 generations, with a15.6% increase in the strongest line (FNH2-2).The high oil trait of the pooled segregating T2 generation was stable and heritable, as it was similar to the average of the corresponding homozygous T3 progeny (compare Fig 5A with S2B Fig). Combining the results from the T2 and T3 lines, these data indicate that *AhLPAT2*promotes the accumulation of seed oil. Taken together with the increase in seed yield per plant in the transgenic lines, the overall seed oil production per plant in the *Napin*: *AhLPAT2* lines increased by approximately 9.5% in theFNH1-21 line and by 43.5% in the FNH2-2 line compared with the wild-type plants (p<0.05; Fig 5B); this increase was due to the higher seed yield per plant and the increased seed oil content. However, the opposite result was obtained for the protein levels in the *AhLPAT2* seeds(Fig 5C); presumably, the increased oil biosynthesis in the transgenic seeds may decrease the carbon flux to the protein biosynthetic pathways.

### FA levels and composition in mature *AhLPAT2* transgenic Arabidopsis seeds

**Fig 6:**
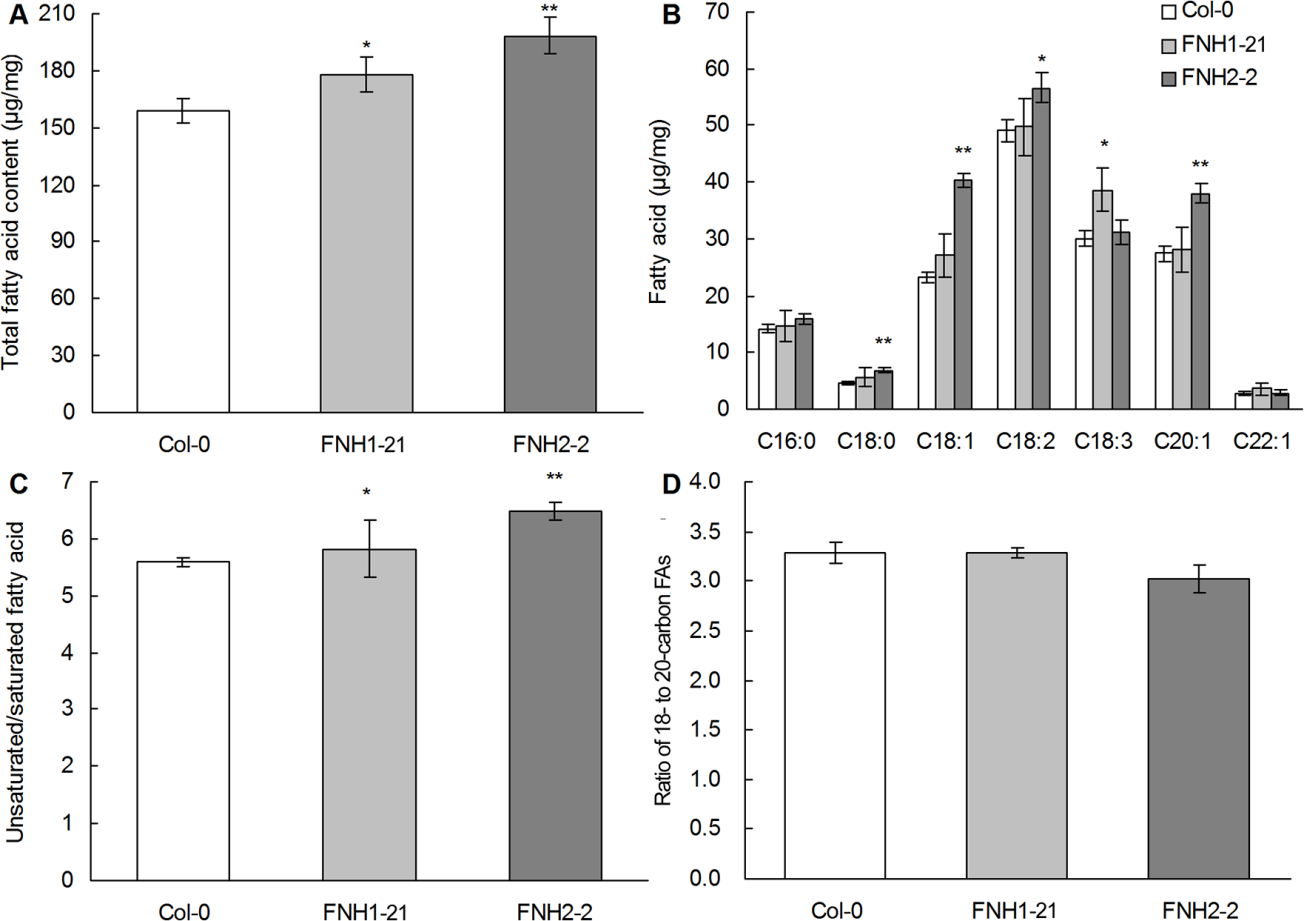
Effect of *AhLPAT2* Overexpression on FA Content and Composition in Independent Homozygous Seeds. A. Total FA content. B. Main FA profiles. C. The ratio of unsaturated to saturated FAs. D. The ratio of 18- to 20-carbon FA. Values are means ± SE (*n* = 10). Asterisks indicate a significant differences between the wild-type and transgenic lines at p < 0.01 (**) and p < 0.05 (*).

We measured the FA content of whole mature seeds in the*AhLPAT2* transgenic Arabidopsis lines. Compared with the corresponding wildtype, the total FA content of the seeds increased by 9.7% to 20.5% in T2 overexpressing seeds and by 11.8% to 24.6% in seeds of the corresponding T3 homozygous *Napin*:*AhLPAT2* lines (S3 Fig and Fig 6A).

To determine if the increase in the total FA content in the seeds of the overexpression lines was derived from an increase in one or many specific FAs, we analyzed the changes in abundance of the major FAs in the transgenic seeds. We found that most of the FAs examined(except 22:1)accumulated to higher levels in the transgenic T2 and T3 seeds than in the wild-type seeds. The changes in FA composition in the T2 seeds were similar to those in the T3 homozygous seeds. The FAs with the greatest increases in the transgenic seeds were 18:0, 18:1, 18:2 and 18:3. The levels of the longer chain FA20:1 increased to a lesser degree (S4A Fig and Fig 6B).However, the changes of 18:3 showed characteristic differences between the transgenic T2 and T3 seeds in the overexpression lines. The 18:3 content in FNH2-2 line T3 seeds was not altered as compared with the wild type, while the amount of 18:3 was significantly increased by approximately 70% in transgenic T2 seeds compared with the wild type (Fig 6B and S4A Fig). This result needs to be confirmed by an exhaustive analysis of TAG molecular species. These results suggested that the seed-specific overexpression of *AhLPAT2*can change the FA composition of the seeds.

Seed oil quality depends primarily on the chain length and saturation of the FAs. The FA saturation was significantly altered in the transgenic seeds. The saturated FA level increased slightly in the*AhLPAT2*transgenic seeds. By contrast, the unsaturated FA level in the seeds of the two lines was significantly greater than that of the wild-type seeds. Therefore, the ratio of unsaturated to saturated FAs increased by more than 10% in the transgenic T3 seeds compared withthe wild-type seeds (p<0.05; Fig 6C).The most common fatty acyl carbon chain lengths in seed TAGs were18C and 20C. We analyzed the changes in the FA carbon chain lengths in the transgenic seeds and found that the ratio of 18Cto 20CFAs either did not change significantly or decreased slightly(Fig 6D).Similar results were obtained in both the T2 and T3 generations of single T-DNA insertion lines (S4B and S4C Fig).Taken together, these results indicate that *AhLPAT2* is associated with altered proportions of unsaturated FAs in storage TAGs but not with changes in the lengths of the carbon chains.

### Differential expression of representative FA and TAG biosynthetic genes in transgenic *AhLPAT2* Arabidopsis siliques

**Fig 7:**
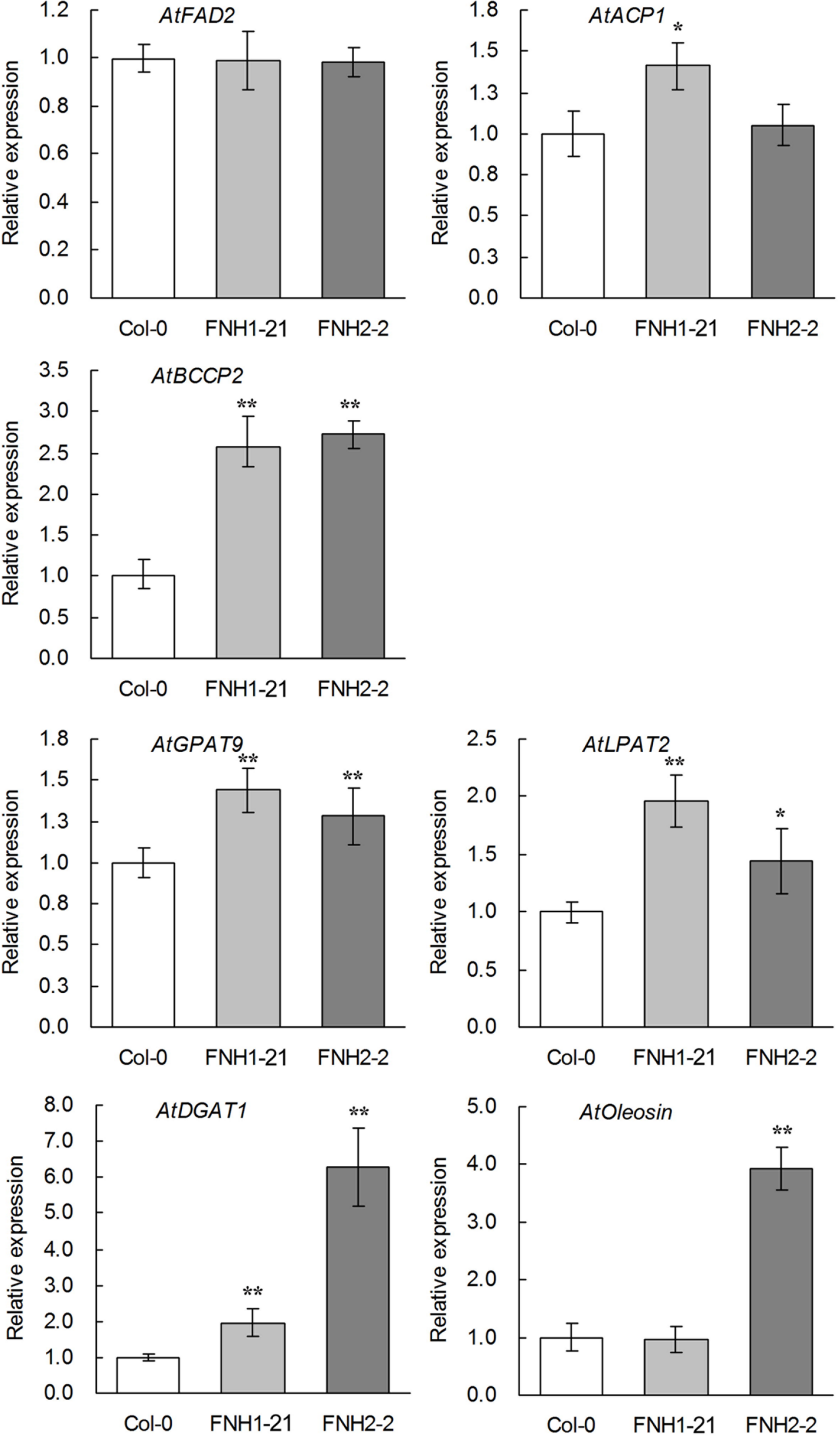
Altered Expression of Genes Involved in the FAs and TAG Biosynthesis Pathway. Values are means ± SE (*n* = 3). Total RNA was prepared from developing siliques of transgenic lines. Gene expression levels are shown relative to the expression of *AtActin7* in each sample. The transcription level of each gene in the wildtype was set as 1.0.Asterisks indicate significant differences between the wild-type and transgenic lines at p < 0.01 (**) and p < 0.05 (*).

To gain insight into the molecular mechanism by which *AhLPAT2*facilitatesFA biosynthesis and TAG assembly, we measured the expression levels of several representative FA and TAG biosynthetic genes in developing transgenic Arabidopsis siliques. These genes included key genes in the fatty acid synthetic pathway(*FAD*, *ACP*, and *BCCP*) and the Kennedy pathway (*GPAT*,*LPAT*, and *DGAT*), as well as the gene encoding the major oil body protein(*Oleosin*).During FA synthesis, delta-12 fatty acid desaturase (FAD2) is the main enzyme responsible for polyunsaturated lipid synthesis in developing seeds; it catalyzes the conversion of 18:1 to 18:2. The expression of *AtFAD2* was the same in transgenic and wild-type siliques (Fig 7), indicating that the increased 18:2 content associated with *AhLPAT2* was not caused by elevated expression of the gene encoding the enzyme that directly participates in FA desaturation. Acyl carrier protein(ACP)carries acyl intermediates and plays a central role in FA biosynthesis [53, 54]. Biotin carboxyl carrier protein(BCCP) is a subunit of ACCase; in the first committed step of FA biosynthesis, which is also the rate-limiting step[55, 56], it serves as a carrier protein for biotin and carboxybiotin for the carboxylation of acetyl-CoA to form malonyl-CoA [57, 58]. *AtACP1* and *AtBCCP2*were analyzed in young T3 siliques at 14 DAF. The transcript levels of the *AtACP1* and*AtBCCP2* genes were higher in the transgenic siliques (Fig 7), which can likely be ascribed to the increased total FA level in the transgenic seeds.

In the Kennedy pathway, G3P is sequentially acylated by G3P acyltransferase (GPAT), LPAT and DAG acyltransferase(DGAT). We noticed that the transcript levels of four related TAG biosynthesis genes in the Kennedy pathway, including *AtGPAT9*, *AtLPAT2*, and *AtDGAT1*, were up-regulated to varying degrees in the transgenic lines (Fig 7), which may be consistent with the increased TAG levels in the *AhLPAT2*-overexpressing seeds. There are four *AtOleosin* isoforms in Arabidopsis. Compared with the wildtype, the mRNA level of *AtOleosin* increased nearly3.9-fold in the FNH2-2 siliques(Fig 7).In FNH1-21 transgenic seeds, despite the low expression of *AtOleosin*, the TAG level was elevated, possibly due to the action of the other *AtOleosin* isoforms.

### Increased expression of key genes involved in sucrose metabolism and glycolysis in transgenic *AhLPAT2* Arabidopsis siliques

**Fig 8:**
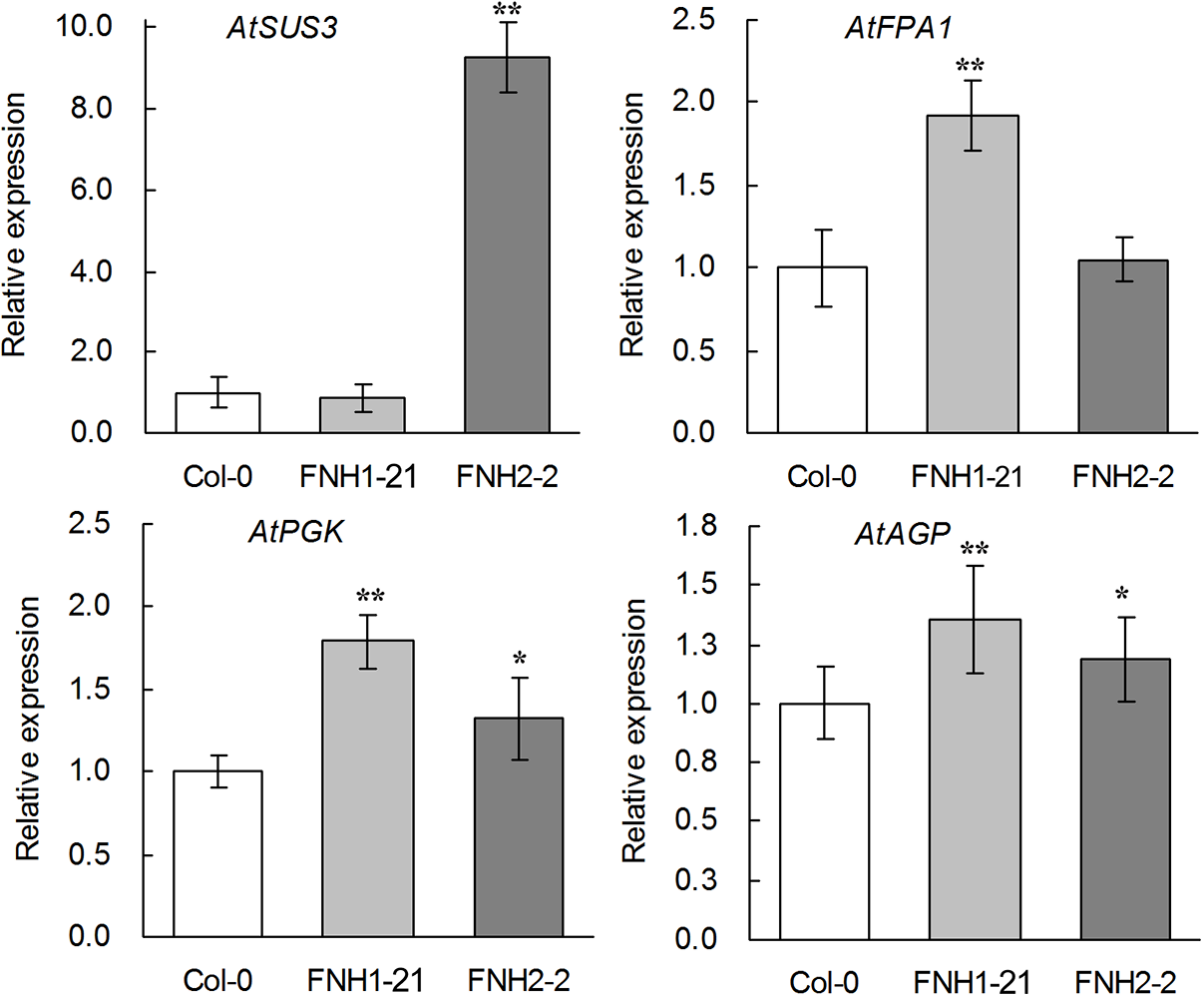
Altered Expression of Genes Involved in Sucrose Metabolism and Glycolysis. Values are means ± SE (*n* = 3). Total RNA was prepared from developing siliques of transgenic lines. Gene expression levels are shown relative to the expression of *AtActin7* in each sample. The transcription level of each gene in the wild type was set as 1.0.Asterisks indicate significant differences between the wild-type and transgenic lines at p < 0.01 (**) and p < 0.05 (*).

Many previous studies have pointed out that biosynthesis of FA and TAG is tightly linked to photosynthesis, carbohydrate (sucrose) metabolism, and glycolysis which provide carbon source for FA synthesis[34, 59, 60]. It has been found that intermediates of sugar metabolism such as glucose-6-phosphate (Glc6P) and pyruvate are imported into plastid from the cytoplasm [61]. Pyruvate dehydrogenase complex in plastids can transform pyruvate into acetyl-CoA, which is the substrate for *de novo* synthesis of FA. Besides acetyl-CoA, sugar metabolism such as the pentose phosphate pathway also provides reducing power for FA biosynthesis, thus indicating that sugar metabolism is highly bound to FA biosynthesis. We attempted to detect whether the expression levels of genes involved in sucrose photoassimilation and glycolysis were altered. Sucrose synthase (SUS) plays a central role in photosynthetic carbon assimilation and partitioning. In the glycolytic pathway, fructose-bisphosphate aldolase (FPA) is an essential enzyme that catalyzes a reversible aldol reaction, and phosphoglycerate kinase (PGK) is a major enzyme used in the first ATP-generating step of the glycolytic pathway. ADP-Glc pyrophosphorylase (AGP) catalyzes the conversion of Glc1P to ADP-Glc, which is then incorporated into starch granules in developing seeds and acts as a regulatory enzyme for plant starch synthesis [62, 63]. In the developing siliques of the *AhLPAT2* transgenic plants, the expression of *AtSUS3* was highly or slightly increased compared with the wildtype (Fig 8). Compared with the wildtype, the mRNA abundance of *AtFPA1* and *AtPGK* was at least 1.5-fold higher in the transgenic siliques (Fig 8).The mRNA level of *AtAGP* was also significantly higher in the transgenic lines (Fig 8).These data demonstrate that the mRNA levels of genes involved in sucrose metabolism, glycolysis, and starch synthesis are increased in the transgenic *AhLPAT2*-overexpressing Arabidopsis plants.

## Discussion

Our results indicate that peanut *AhLPAT2* plays important roles in the seed oil accumulation pathway and provide clues to understanding of the molecular mechanisms controlling oil biosynthesis and storage in peanut seeds. In agreement with previous results, our current data indicate that the transcript abundance of *AhLPAT2*was higher in the seeds of a high-oil peanut cultivar than in a low-oil cultivar between 40 and 60 DAF. These patterns are consistent with a proposed general role for TAG metabolism in seeds. Similar to how quantitative trait loci (QTLs) and transcriptomics can be used to identify genes in the seed oil accumulation pathway[38, 64], our experimental materials with large differences in their seed oil content make it easier to identify differentially expressed genes associated with seed oil content. It has been reported that in developing castor seeds, the *RcLPATB*gene[31]grouped with members of the so-called B-class LPAT enzymes and the*RcLPAT2* gene[65];these genes are highly induced during endosperm expansion and maturation, when oil biosynthesis and accumulation are maximal.

We analyzed the subcellular localization of AhLPAT2 in peanut. Inspection of the subcellular localization of peanut AhLPAT2 in onion epidermal cells revealed that this protein is distributed rather randomly in the cytosol and is not concentrated on any membranes structures such as ER(Fig 2E and 2H). Analysis of amino acid sequences of AhLPAT2 by PSORT tool revealed that the AhLPAT2 protein lacked a nuclear localization signal in its sequence[33], which is consistent with the localization of AhLPAT2 in the cytosol. In contrast, five AtLPAT1 to AtLPAT5 identified from Arabidopsis so far are membrane bound through transmembrane domains, especially AtLPAT2, which is ubiquitous and ER located[21]. At4g24160, the first soluble LPAT in plants, was found to be localized in the mitochondria and the chloroplast[66].However, our results do not clarify, whether AhLPAT2 is actually present in the same cells in developing peanut seeds, flowers, or leaves or whether *AhLPAT2* expression is restricted to different tissue types within these organs. These questions will need to be addressed using mono-specific AhLPAT2 antibodies and immunomicroscopic analysis of native peanut tissues[67].Regardless, it is tempting that *AhLPAT2* has been shown to play a role in the production of TAGs during seed development in transgenic plants.

*AhLPAT2* overexpression using a seed-specific promoter enhanced oil content, FA accumulation, and seed weight in Arabidopsis transgenic plants. Nevertheless, in our present study, the two transgenic lines selected show markedly different effects on expression levels of *AhLPAT2* and endogenous genes, seed yield, seed oil content, oil yield, and seed FAs content. It is possible that the *AhLPAT2*fragmentmay be integrated into different locus of the chromosomal DNA of the two transgenic Arabidopsis lines. Furthermore, recent studies have revealed that the integration sites of the exogenous genes were unpredictable[68]. The site effect and the structure of the exogenous genes have significant effects on the gene expression and phenotype differences of the transgenic plants. In this study and under our experimental conditions, transgenic overexpression of *AhLPAT2* resulted in a 12.2% increase in oil content and an 11.8% to 24.6% increase in total FA content in T3 Arabidopsis seeds without changing other agronomic traits. The change in the protein content of the transgenic *AhLPAT2* seeds had the opposite effect. In addition to the seed quality traits, the seed yield-related traits were examined in the*AhLAPT2* lines. An analysis of seed weight suggested that the overexpression of *AhLPAT2* leads to a significant or slight increase in the average seed mass. The total seed yield in milligrams per plant increased by0.3% to 24.4%. We hypothesize that the increase in seed weight could be the result of increased FA and oil accumulation in transgenic seeds[39]. These current results corroborated several previous predictions derived from our retrospective data [33]. Recent studies have also shown that the LPAT-catalyzed conversion of LPA to PA is a metabolic pivotal point in the provision of intermediates required for the synthesis of diverse glycerolipids including membrane lipids and neutral TAG[23]. Apparently, an increase in LPAT activity can result in the enlargement of the storage lipid sink through a feed-forward effect.

In addition, the above findings from our study are also consistent with the results from other studies that show that LPAT mainly catalyzes TAG synthesis for seed storage,except for participation in the synthesis of membrane lipids in eukaryotes[21, 69]. Many studies have demonstrated that *LPAT* overexpression in the model plant Arabidopsis leads to a significant increase in seed oil content. When *SLC1*and *SLC1-1*[27] were expressed in Arabidopsis, the seed oil content rose from 8% to 48%, showing that the TAG content of the seeds had increased [26]. The overexpression of rapeseed *LPAT* isoforms (*BAT1*.*13* and *BAT1*.*5*) with homology to Arabidopsis *AtLPAT2* in Arabidopsis seeds also resulted in 6% and 13% increases in average seed weight and total seed FA content, respectively, compared with non-transformed plants [23]. By contrast, reducing the expression of the homologous Arabidopsis *LPAT* gene resulted in a 30% lower seed mass and a 16% lower TAG content, frequently resulting in a wrinkled seed phenotype [70]. This study shows that the individual line with the largest change in transcript abundance also had the highest percentage of seed oil content and total oil yield per plant. Our results are consistent with the findings of previous studies [71, 72], which show a correlation between *AhLPAT2* transcript abundance and oil content but do not provide a mechanism for the relationship between the increase in expression of *AhLAPT2*and the increase in LPAT activity. However, several mechanisms have been proposed in which Arabidopsis DGAT catalyzes the last step of TAG formation[39], lending support to our present study.

An increase in seed oil content in transgenic Arabidopsis plants can lead to an increase in plant oil yield under the conditions used in this study. Most genetic studies and breeding programs for oilseed crops focus on increasing the amount of oil produced by a plant[73]. Presently, we found that the total oil yield (milligrams per plant) was significantly increased (p<0.05) in the *AhLPAT2* lines, with a maximum increase of 43.5% in the best transgenic line compared with the wild-type control. The increase in seed oil production is mainly due to an increase in seed oil content and seed weight without a significant change (p>0.05) in seed number per plant, similar to the case of rapeseed microsomal *LPAT*s expressed intransgenic Arabidopsis[23]. Similar mechanisms of lipid accumulation may have been affected by rapeseed *LPAT*s and peanut *AhLPAT2*, although further studies are needed to confirm this.

As expected, the results derived from Arabidopsis are also consistent with the results from other studieson oilseed crops. For instance, *SLC-1*was introduced into a rapeseed cultivar with high erucic acid (22:1^ω9^) levels [26]. The resulting transgenic line showed a substantial increase in seed oil content and an increase in the proportion of erucic acid. The transgenic line was later tested in the field and exhibited a 53% to 121% increase in total erucic acid yield (weight/plot) [74], and the six best T5 field-grown lines showed a 2.8% to 5.6% increase in oil content, representing overall net increases in oil content of 6.7% to 13.5% on a dry-weight basis [75]. In soybean, the *SLC1* gene was expressed in somatic embryos under the control of the seed-specific phaseolin promoter. Some transgenic somatic embryos in both T2 and T3 transgenic seeds showed an average increase in oil content of 1.5% over the controls, with a maximum increase of 3.2% in one T3 line [27]. Taken together, these results suggest that increasing the expression of *LPAT* in seeds may promote the flow of carbon intermediates into TAG through the Kennedy pathway, resulting in increased TAG accumulation; these observations may be of value for increasing lipid yield via transgenesis [70].It is possible that *AhLPAT2* would promote lipid accumulation in peanut seeds as it did in transgenic Arabidopsis seeds, although more studies are needed to determine this result. Above all, these data indicated that improvement in seed quality traits in peanut may be achieved by the manipulation of the *AhLPAT2* expression level. This manipulation may improve genetics not only for peanut but also for other oil crops.

We found that the*AhLPAT2* gene could preferentially incorporate unsaturated FAs into lipids from Arabidopsis seeds. However, AhLPAT2 seems to lack a preference for the length of the FA carbon chain. This finding is consistent with those of other studies on diverse traditional oilseed crops, which suggest that LPAT, and especially microsomal LPAT, has a general preference for 16- and 18-carbon monounsaturated FAs over saturated FAs[65].In Arabidopsis, AtLPAT2 was expected to preferentially utilize 18:1-CoA over other fatty acyl-CoAs [21]. In addition, a similar result was found for LAT1 in *Limnanthes*, whereas LAT2 showed a broader specificity, with 16:0-CoA being incorporated at a rate that was even higher than that of 18:1-CoA [76].The EpLPAT2 enzyme of *Echium pitardii* displays high activity for 18:3*n*3-CoA and 18:2*n*6-CoA [25]. It has been shown that the acyl-CoA specificity of castor bean RcLPAT2 is strongly influenced by the acyl group at the *sn*-1 position of the acceptor LPA substrate [31]. In addition, special LPATs that can incorporate unusual acyl groups are found in the developing seeds of certain plant species, such as *Limnanthes* and coconut[22].However, many other studies pointed to the opposite conclusion. For example, the FA composition of the TAG fraction in *BAT1*.*13* and *BAT1*.*5* transgenic plants did not vary significantly in the proportions of the major classes of FAs or in the very-long-chain-FAs (20:1-CoA and 22:1-CoA) compared with the wild-type control plants[23].Additionally, Brown, Slabas [29]also found that in the majority of species examined, there is no correlation between the final *sn*-2 composition of the oil and the observed selectivity of the LPAT enzyme. Similarly, Roscoe [70]reported that the FA composition of transgenic lines was minimally affected by overexpression of the yeast *SLC1*gene or the homologous Arabidopsis *LPAT* gene. We speculate that this discrepancy could be due to differences between species types. Although our data strongly favor a role for *AhLPAT2* in regulating FA composition, the acyl-CoA pool composition of the cell may also play a role in particular plants because the FA composition of TAG depends on both the substrate specificity of the acyltransferases and the acyl-CoA composition of the cell [77].There is a need for further detailed studies and verification of the specificity of the AhLPAT2 enzyme using LPA and oleoyl-CoA as substrates.

In this work, the transcript levels for multiple selected ACCase and acyltransferase genes were increased in developing Arabidopsis siliques of *AhLPAT2* seed-specific overexpression lines, thereby influencing FA and TAG accumulation. For example, the mRNA abundances of *AtBCCP2* and *AtACP1*were increased in overexpression lines compared with the wild type, which are involved in *de novo* FA biosynthesis. It seems likely that an increase in lipid accumulation in seeds requires upregulation of multiple related genes involved in carbon metabolism, FA synthesis, and end-product synthesis of TAGs from FAs. It has been proposed that expression of transgenes encoding plant enzymatic functions can interfere with the normal expression patterns of corresponding endogenous genes as well as genes involved in the same or related processes. Although it is possible that the phenotype observed in overexpressing *AhLPAT2* transgenic plants is due to solely to increased activity of this one enzymatic step, it is conceivable also that altering AhLPAT2 activity results in upstream metabolic pathways contributing to the biosynthesis of FA, to be incorporated into TAG. Moreover, we speculated that increases in AhLPAT2 activity may lower the size of the acyl-CoA pools, thereby signaling a need for enhanced FA synthesis. Together, our data suggest that increasing AhLPAT2 activity affects a series of genes involved in lipid metabolism, possibly through feedback or feed-forward effects[55].However, qRT-PCR analysis showed that peanut *AhLPAT2* did not strongly induce the expression of endogenous Arabidopsis *AtFAD2*. This finding suggests that *AhLPAT2* may not have a strong effect on the*AtFAD2* gene. Alternatively, another mechanism may also be involved in the *AhLPAT2*-dependent TAG assembly processes. Consistent with these results, the content of almost every major FA, not just 18:2, was higher in the transgenic seeds. Surprisingly, our results indicated that several important genes that directly participate in TAG assembly via the Kennedy pathway, such as *AtGPAT9AtLPAT2* and *AtDGAT1*,were also expressed at higher levels in the *AhLPAT2* overexpressing lines compared with wild-type plants. Similar results were observed in transgenic rapeseed [78]and potato [79] overexpressing *CaLPAT* from *Crambe abyssinica*. However, the mechanism by which this phenomenon works in plants remains elusive; additional studies are needed to determine if the observed increase in storage lipids is caused by the exogenous *AhLPAT2*. We hypothesize that the interaction between exogenous *AhLPAT2* and endogenous acyltransferase-encoding genes may have an additive effect on the percent seed oil content.

Previous studies have shown that when individual SUS isoforms are eliminated in pea [80] and maize [81], the seed weight and composition in these species are markedly changed. In this study, the expression of *AtSUS3* was considerably induced in the transgenic lines. These observations, combined with our results and the molecular mechanism of the source-sink network in plants[82, 83], suggest that there is positive feedback between sucrose and lipids. In contrast to *AtSUS3*, the transgenic lines exhibited only moderate changes in the relative expression of key glycolytic genes such as*AtFPA1*, *AtPGK* and *AtAGP*. It is not surprising that increases in *LPAT* transcript abundance or activity may reduce the size of the acyl-CoA pool, thereby signaling a need for enhanced FA synthesis through, for instance, the promotion of ACCase activity[39].

In summary, in peanut, although several QTLs that influence seed oil content have been mapped, the specific genes associated with these QTLs have not yet been identified[6, 84]. Inthe present study, a novel *AhLAPT2* gene from peanut was cloned, and its functions wereanalyzed. Our results show that seed-specific overexpression of *AhLPAT2* has the potential to increase Arabidopsis seed oil content and weight. Analysis of seed yield per plant in transgenic Arabidopsis indicated that the total oil yield is also significantly increased. The plants had more unsaturated FAs and higher total FA content. In the siliques of homozygous transgenic lines, the relative expression levels of several genes involved in FA biosynthesis, TAG assembly, sucrose metabolism and glycolysis were also significantly increased. Therefore, *AhLPAT2* is of interest for the genetic engineering of seed oil and FA composition and especially for decreasing the saturated FA content of edible vegetable oils.

## Supporting Information

S1 Fig Effect of *AhLPAT2* Overexpression on Arabidopsis Plant Development. A. Plant height of mature Arabidopsis. B. Mature silique length. C. Mature seed size. Values are means ± SE of measurements on individual plants (*n* = 10).
(TIF)

S2 Fig Oil Content Analysis of Transgenic T1 and T2 Generation Transgenic Arabidopsis Seeds. A. Oil content distribution of transgenic T1 seeds. Col-0, wild-type control including 11 plants; FNH T1, Transgenic T1 Arabidopsis including 42 plants. The box contains 50% of the data points. The bars across boxes represent the medians. The top and bottom ends of the ‘whiskers’ represent the highest and lowest values observed. Black dots represent outliers. B. Seed oil content of homozygous T2 *AhLPAT2* transgenic Arabidopsis plants. Seed oil content was determined by the NMR method. Mean FNH1 T2 indicates the mean of five FNH1 transformants; Mean FNH2 T2 indicates the mean of six FNH2 transformants; Mean FNH T2 indicates the mean of eleven FNH1 and FNH2 transformants. Values are average seed oil percentage ± SE (*n* = 5 and 6 for FNH1 and FNH2, respectively). Asterisks indicate significant differences between the wild-type and transgenic lines at p < 0.01 (**) and p < 0.05 (*).
(TIF)

S3 Fig The Effect of *AhLPAT2* Overexpression on Total FA Content in Independent T2 Homozygous Seeds. The values are means ± SE (*n* = 20). Asterisks indicate significant differences between the wild-type and transgenic lines at p < 0.01.
(TIF)

S4 Fig The Effect of *AhLPAT2* Overexpression on FA Profiles in Independent T2 Homozygous Seeds. A. The main FA composition. B. The ratio of unsaturated to saturated FAs. C. The ratio of 18- and 20-carbon FAs. The values are means ± SE (*n* = 20). Asterisks indicate significant differences between the wild-type and transgenic lines at p < 0.01 (**) and p < 0.05 (*).
(TIF)

S1 Table Arabidopsis Gene-specific qRT-PCR Primers Used in this Study. (DOC)
