# Supplementary figures and images for: The Peanut (Arachis hypogaea L.) Gene AhLPAT2 Increases the Lipid Content of Transgenic Arabidopsis Seeds
Source: PLoS One. 2015 Aug 24;10(8):e0136170. doi: 10.1371/journal.pone.0136170 (PMC4547709; doi:10.1371/journal.pone.0136170)

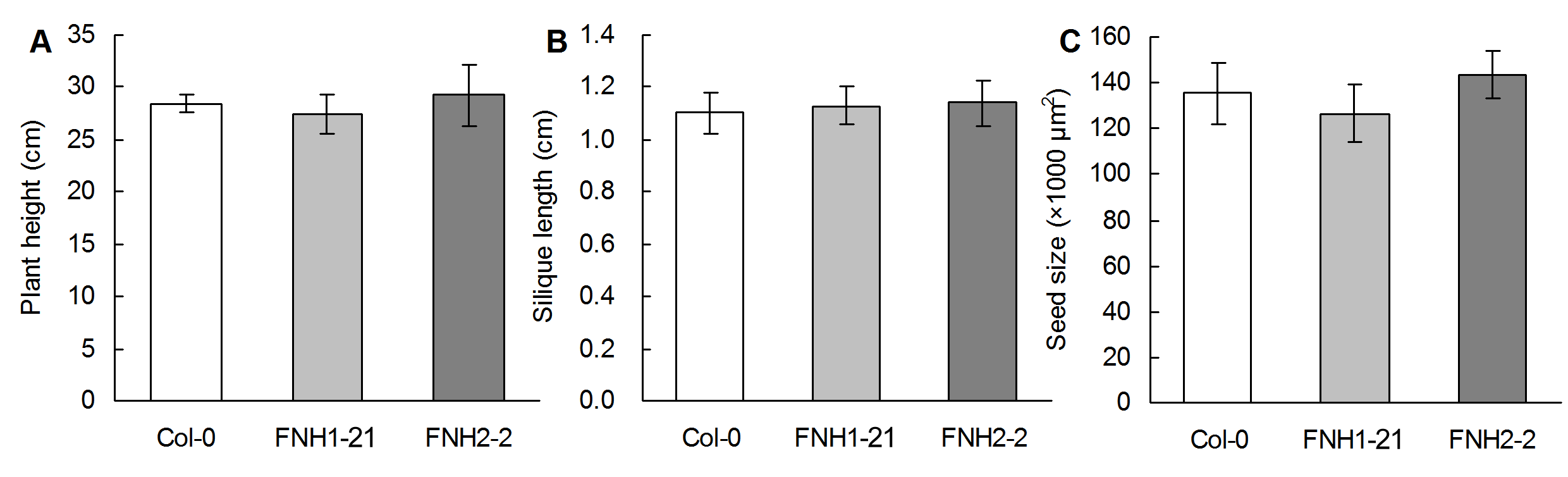

Supplement: S1 Fig — A. Plant height of mature Arabidopsis. B. Mature silique length. C. Mature seed size. Values are means ± SE of measurements on individual plants (n = 10). (TIF) [file pone.0136170.s001.tif]

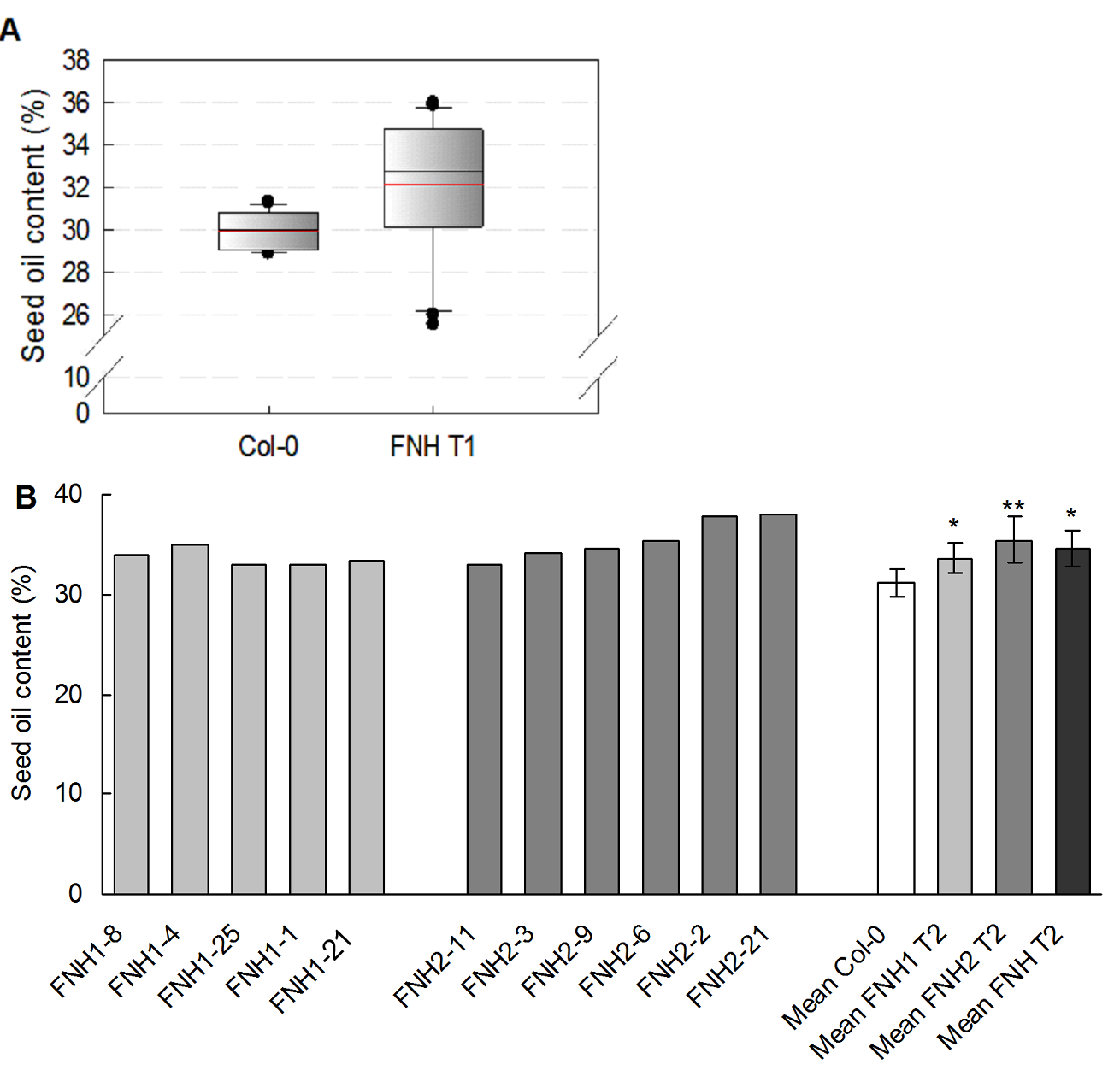

Supplement: S2 Fig — A. Oil content distribution of transgenic T1 seeds. Col-0, wild-type control including 11 plants; FNH T1, Transgenic T1 Arabidopsis including 42 plants. The box contains 50% of the data points. The bars across boxes represent the medians. The top and bottom ends of the ‘whiskers’ represent the highest and lowest values observed. Black dots represent outliers. B. Seed oil content of homozygous T2 AhLPAT2 transgenic Arabidopsis plants. Seed oil content was determined by the NMR method. Mean FNH1 T2 indicates the mean of five FNH1 transformants; Mean FNH2 T2 indicates the mean of six FNH2 transformants; Mean FNH T2 indicates the mean of eleven FNH1 and FNH2 transformants. Values are average seed oil percentage ± SE (n = 5 and 6 for FNH1 and FNH2, respectively). Asterisks indicate significant differences between the wild-type and transgenic lines at p < 0.01 (**) and p < 0.05 (*). (TIF) [file pone.0136170.s002.tif]

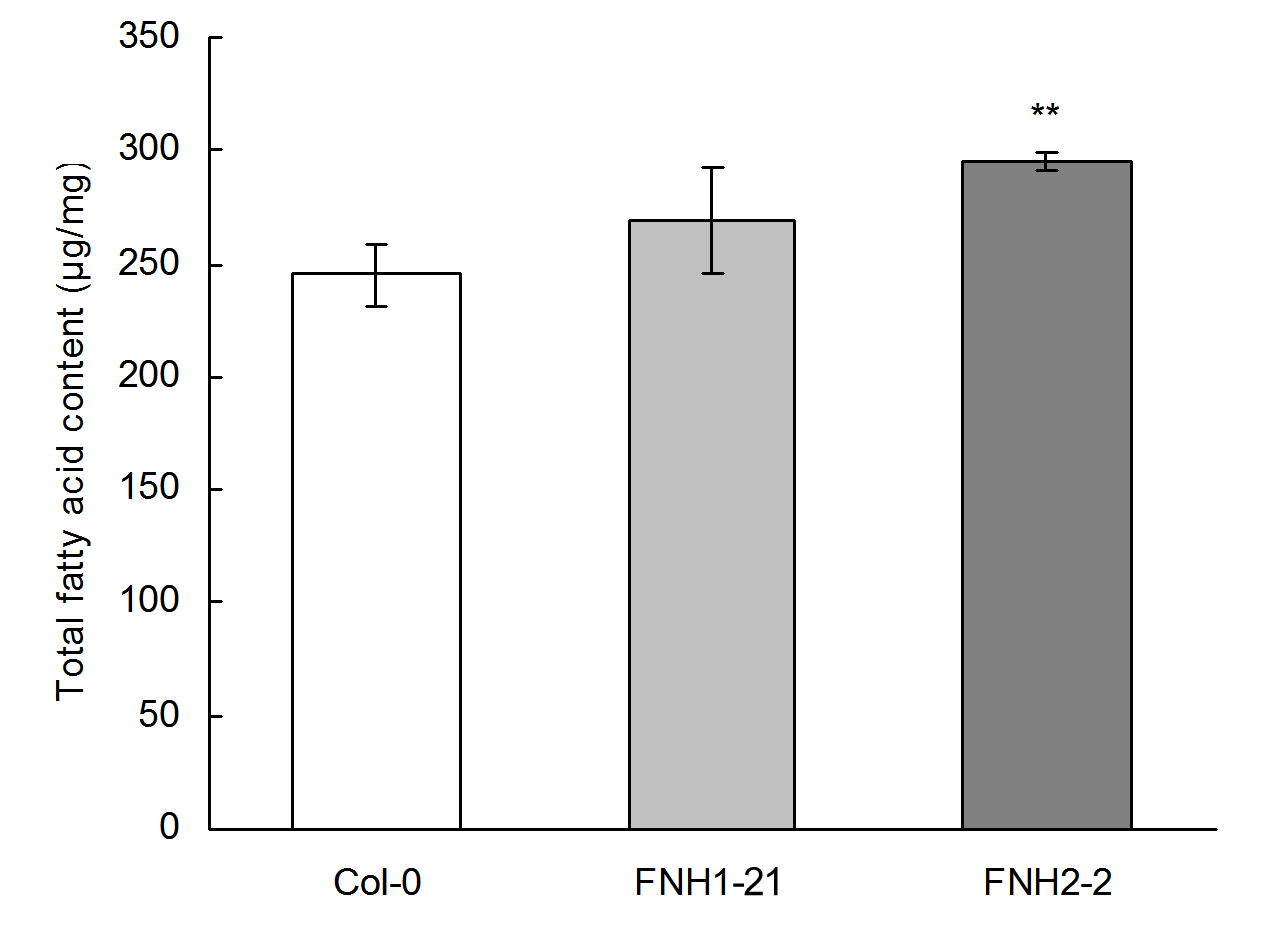

Supplement: S3 Fig — The values are means ± SE (n = 20). Asterisks indicate significant differences between the wild-type and transgenic lines at p < 0.01. (TIF) [file pone.0136170.s003.tif]

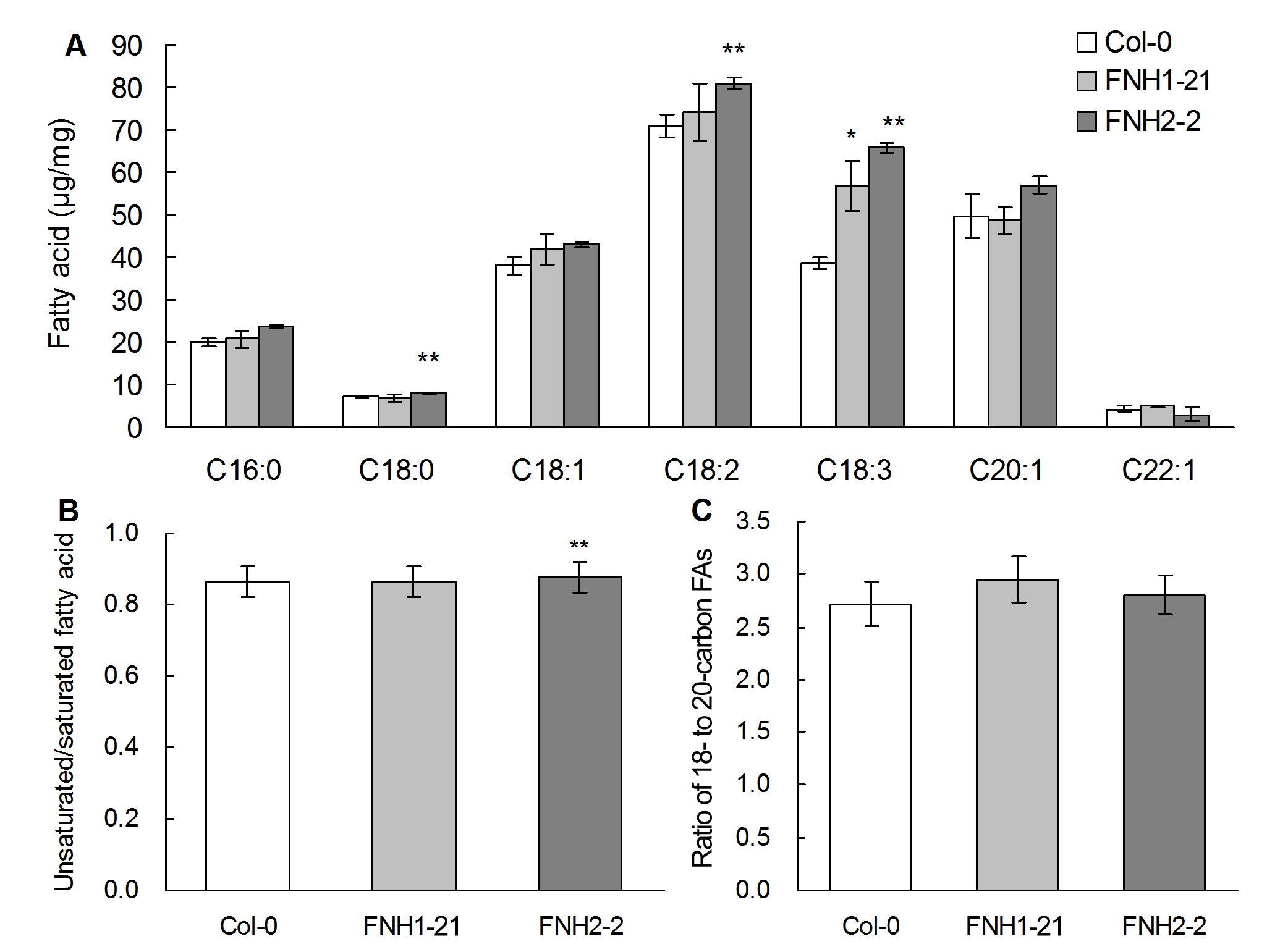

Supplement: S4 Fig — A. The main FA composition. B. The ratio of unsaturated to saturated FAs. C. The ratio of 18- and 20-carbon FAs. The values are means ± SE (n = 20). Asterisks indicate significant differences between the wild-type and transgenic lines at p < 0.01 (**) and p < 0.05 (*). (TIF) [file pone.0136170.s004.tif]
