# Supplementary material for: The Peanut (Arachis hypogaea L.) Gene AhLPAT2 Increases the Lipid Content of Transgenic Arabidopsis Seeds
Source: PLoS One. 2015 Aug 24;10(8):e0136170. doi: 10.1371/journal.pone.0136170 (PMC4547709; doi:10.1371/journal.pone.0136170)
Supplement: S1 Table — (DOC) [file pone.0136170.s005.doc]

**Table S1.** Arabidopsis Gene-specific qRT-PCR Primers Used in this Study

| Gene name | AGI ID | Primer name | Sequence (5′ to 3′) |
| --- | --- | --- | --- |
| *AtFAD2* | At3g12120 | At.FAD2-F | ATGGGTGCAGGTGGAAGAAT |
|  |  | AtFAD2-R | CCAGGAGAAGTAAGGGACGA |
| *AtACP1* | At3g05020 | AtACP1-F | AATCTATCCTTCAACCTCCGCCGT |
|  |  | AtACP1-R | AGAGAATCTGCTCCAAGGTCAGCA |
| *AtBCCP2* | At5g15530 | AtBCCP2-F | AACCCAATGGGATCTCCTTTCCCT |
|  |  | AtBCCP2-R | ATAAATTCAGAGAGCTCGGCGGGT |
| *AtOleosin* | At3g01570 | AtOleosin-F | TCCTTTATCCTCAGAGTGGCCCTT |
|  |  | AtOleosin-R | AAGGCTGCTGGTACAATAACCGGA |
| *AtSUS3* | At4g02280 | AtSUS3-F | GAGATACCGCAGGGAGAGTT |
|  |  | AtSUS3-R | CAGCATTTCAGTCTCAAGGG |
| *AtFPA1* | At2g21330 | AtFPA1-F | CACCGCTGCTTACTACCAAC |
|  |  | AtFPA1-R | TTCTCCGTCCAACATAATCTCTG |
| *AtPGK* | At1g79550 | AtPGK-F | TGCTGCTGGAACTGAGGCCG |
|  |  | AtPGK-R | GCGAGGACTCCTGGAAGTGGC |
| *AtAGP* | At5g19220 | AtAGP-F | CGCCAAGCCTGCCGTTCCTA |
|  |  | AtAGP-R | TGGCGTTTGAGTGGCCGCAA |
| *AtGPAT9* | At5g60620 | AtGPAT9-F | TCGGAAACCGGCGACGTAAGC |
|  |  | AtGPAT9-R | TGGCACCAGCAGCTTCAGTGAG |
| *AtDGAT1* | At2g19450 | AtDGAT1-F | TGGAGCTCCCGCCGACGTTA |
|  |  | AtDGAT1-R | TCGCCCTCCGATGAGCTGGA |
| *AtLPAT2* | At3g57650 | AtLPAT2-F | CCGGGTGGTTGCAGAAACCTTGT |
|  |  | AtLPAT2-R | TCCCAGGCAACCTGACCGCT |
| *AtActin7* | At5g09810 | AtActin7-F | GGAACTGGAATGGTGAAGGCTG |
|  |  | AtActin7-R | CGATTGGATACTTCAGAGTGAGGA |
